# Gas Porosimetry by Gas Adsorption as an Efficient Tool for the Assessment of the Shaping Effect in Commercial Zeolites

**DOI:** 10.3390/nano11051205

**Published:** 2021-05-01

**Authors:** Alejandro Orsikowsky-Sanchez, Christine Franke, Alexander Sachse, Eric Ferrage, Sabine Petit, Julien Brunet, Frédéric Plantier, Christelle Miqueu

**Affiliations:** 1 TOTAL EP—Pôle d’Etudes et de Recherche de Lacq (PERL), BP 64170 Lacq, France; 2 Laboratoire des Fluides Complexes et leurs Réservoirs, Université de Pau et des Pays de l’Adour, E2S UPPA, CNRS, 64600 Anglet, France; frederic.plantier@univ-pau.fr; 3 MINES ParisTech, Center of Geosciences, PSL University, CEDEX, 77305 Fontainebleau, France; christine.franke@mines-paristech.fr; 4 Université de Poitiers—IC2MP, UMR 7285 CNRS, 86073 Poitiers, France; alexander.sachse@univ-poitiers.fr (A.S.); eric.ferrage@univ-poitiers.fr (E.F.); sabine.petit@univ-poitiers.fr (S.P.); julien.brunet@univ-poitiers.fr (J.B.)

**Keywords:** zeolite, binder, CO_2_, gas porosimetry, adsorption

## Abstract

A set of three commercial zeolites (13X, 5A, and 4A) of two distinct shapes have been characterized: (i) pure zeolite powders and (ii) extruded spherical beads composed of pure zeolite powders and an unknown amount of binder used during their preparation process. The coupling of gas porosimetry experiments using argon at 87 K and CO_2_ at 273 K allowed determining both the amount of the binder and its effect on adsorption properties. It was evidenced that the beads contain approximately 25 wt% of binder. Moreover, from CO_2_ adsorption experiments at 273 K, it could be inferred that the binder present in both 13X and 5A zeolites does not interact with the probe molecule. However, for the 4A zeolite, pore filling pressures were shifted and strong interaction with CO_2_ was observed leading to irreversible adsorption of the probe. These results have been compared to XRD, IR spectroscopy, and ICP-AES analysis. The effect of the binder in shaped zeolite bodies can thus have a crucial impact on applications in adsorption and catalysis.

## 1. Introduction

Zeolites are porous materials that have been widely used in industrial applications of adsorption (gas or liquid) and catalysis for more than 60 years [1]. These materials have the major advantage of superior thermal and mechanical stability. Moreover, zeolites are the adsorbents of choice related to their low cost. To study the adsorption mechanisms, adsorbents must be as pure as possible. Thus, adsorbents in powder form (absence of binder) are most suitable. However, in dynamic pilot experiments and industrial processes shaped adsorbents are used to limit the pressure drop. These shaped adsorbents are typically obtained by the extrusion of pure powders through the employment of a binder that provides mechanical strength and cohesion of the individual crystals [1,2,3,4,5]. The most common binders used to shape zeolites are clays (kaolin, metakaolin, attapulgite, bentonite), other inorganic compounds (SiO_2_, Al_2_O_3_), and some organic compounds (carboxymethylcellulose, methylcellulose, lignosulfonate) [1,4]. Yet, in most studies, the impact of the binder on the adsorption mechanisms is scarcely described. Within this limited information, most of the reports focus on the impact of binders in catalysis and in the mechanical properties rather than on adsorption behavior [2,6]. Indeed, Gilson and co-workers very recently observed that binders can react and thus importantly impact acidic properties of zeolites [7]. Some other authors analyzed the effect of the amount of binder on zeolite pore volume [5]. For instance, among the studies that have analyzed the binder effect on gas adsorption, Charkhi et al. [8] investigated the influence of bentonite on the adsorption properties of a granulated nano NaY zeolite through Xenon and Nitrogen adsorption. They showed that increasing the bentonite content from 20 to 40 wt% favors the rupture of load granules by 232% and decreases the BET surface area by 66%. Further, 25 wt% of binder caused a decrease in the Xenon crystal diffusivity. Jasra et al. [9] analyzed the effect of clay binders on the sorption and catalytic properties of mordenite and HY pellets by using the adsorption of N_2_, O_2_, Ar and CH_4_, and X-ray diffraction (XRD). Their study suggests an increase in the surface heterogeneity of zeolites upon pelletization due to the migration of clay cations inside the zeolite cavities. Sun et al. [10] investigated the effect of binders on the adsorption of N-paraffins in 5A zeolite by comparing the amount adsorbed on the pure zeolite and the shaped one through electron microscopy. They concluded that the 5A zeolite with binder shows lower adsorption capacities due to the dilution effect as well as the blocking of pore apertures. More recently, Chen et al. [11] studied the impact of the interactions between a silica binder and a NaY zeolite using XRD, Transmission Electron Microscopy (TEM), and Fourier Transform Infrared (FTIR) spectroscopy techniques, regarding both the structure and the surface effects. They observed that the interactions between the zeolite and the binder, combined with the dealumination of the framework, leads to a decrease in crystal size and crystallinity, and an increase of Si/Al ratio, hydrophobicity, and thermal stability. IR spectra of NH_3_ adsorption on NaY and NaY—SiO_2_ revealed that pelletization caused an increase in the number of Brønsted acid sites. Besides, Cao et al. [12] evaluated the impact of the alumina binder by measuring heats of adsorption of SF_6_ and CO_2_ on silicalite-1 pellets via calorimetric measurements. Their results indicate that the strong adsorption of CO_2_ on alumina binders leads to a significant heterogeneity on the pelletized silicalite-1 sample. Finally, Shams et al. [13] reported the effect of binders composed of kaolin and carboxymethylcellulose (CMC) on the sieving/adsorption properties of a 5A monolith. They characterized the sample using XRD, Scanning Electron Microscopy (SEM), and Energy-dispersive X-ray (EDX) spectroscopy and concluded that the highest ion exchange capacity and the best sieving results were obtained with 30 wt% of binders (kaolin), and that the use of small amounts of CMC has profound effects on the adsorption properties of 5A zeolite.

It becomes clear from these studies that the effect of the binders is not neglectable, in particular if the objective is to study the adsorption mechanisms on shaped samples. The present work aims to propose an efficient control protocol based on gas porosimetry to evaluate the effect of the binder. The comparison of the adsorption of an inert probe molecule such as argon and a molecule with a smaller kinetic diameter and a quadrupole moment such as CO_2_ on both powdered and shaped samples are used for this purpose. The results are compared to XRD, IR spectroscopy, and Induced Coupled Plasma—Atom Emission Spectroscopy (ICP-AES) analysis, not to identify the binder but rather to assess the protocol.

## 2. Materials and Methods

### 2.1. Materials

Three commercial zeolites were considered in this study under two different shapes: pure zeolite powder and spherical beads composed of zeolite powder and a binder of unknown composition and quantity. All zeolite samples were provided by Fisher Scientific (Waltham, MA, USA).

For both 13X and 5A zeolites, two different samples of spherical beads of distinct origin (i.e., two different providers) were compared with one sample of zeolite powder (Table 1). It should be noted that the powder and SB1 samples come from the same provider. In the case of the 4A zeolite, only one shaped sample was available for comparison with the powder sample. The nomenclature adopted in this work is as shown in Table 1.

Argon, helium, and carbon dioxide for gas adsorption experiments were provided by Linde Gas. The first two gases were purchased in 6.0 quality (purity ≥ 99.9999%) and the CO_2_ had a quality of 4.5 (purity ≥ 99.995%).

### 2.2. Method

#### 2.2.1. Gas Porosimetry

All adsorption isotherms were measured using a commercial volumetric apparatus. Argon adsorption isotherms at 87 K were performed using an Autosorb-iQ (Quantachrome Instruments, Anton Paar, Graz, Austria) with the Cryosync^®^ temperature regulation system. Carbon dioxide adsorption isotherms at 273 K were carried out employing an ASAP2020 (Micromeritics Instruments Corporation, Norcross, GA, USA) and an Autosorb-iQ (Quantachrome Instruments, Anton Paar, Graz, Austria). Temperature regulation at 273 K was implemented with an ice-water mixture Dewar and with a double jacket Dewar and a thermostatic bath respectively. To eliminate any trace of gas prior to the experiment, all the samples were degassed during 12 h at 573 K under secondary vacuum (heating ramp of 10 K min^−1^). For further details on these conditions of pretreatment please see Refs [14,15].

#### 2.2.2. X-ray Diffraction Analysis

The XRD patterns were collected using a PANalyticalX’Pert Powder X-ray diffractometer (Malvern Panalytical, Malvern, UK) equipped with a Cu-radiation and an X’celerator detector. The analyses were conducted on homogenized dry bulk samples prepared on randomly oriented powder mounts and were scanned in the 2 to 60°2Ө angular range. The XRD patterns have been interpreted using the EVA© software (Bruker DIFFRAC Plus 2007, version V5; for more details see Giencke [16]). Reference files from the International Centre for Diffraction Data (ICDD PDF2 data files) have been used for mineral phase identification. (Semi) quantification of the present mineral phases was made using the normalized intensity ratio (RIR) method with an uncertainty of ± 5% (e.g., Chung et al. [17]).

#### 2.2.3. Infrared Spectroscopy

Fourier transform infrared (FTIR) spectra were measured in transmission mode at 4 cm^−1^ resolution, in the 4000–400 cm^−1^ range using a Magna-IR 7600 Nicolet spectrometer (ThermoFisher Scientific, Waltham, MA, USA) equipped with an EverGlo source, a KBr beam splitter, and a DTGS-KBr detector. Spectra were measured from KBr pressed pellets dried overnight at 110 °C before measurement to remove adsorbed water. The pellets were prepared by mixing 100 mg of KBr and 0.5 mg (low sample concentration) and 1 mg powdered sample (high sample concentration). The low sample concentration allows avoiding the oversaturation in the strong silicate absorption regions in the 1000–1100 cm^−1^ and 400–550 cm^−1^ regions, while the high sample concentration allows for higher resolution of the small bands. The spectral subtractions were made with the Omnic software (ThermoFisher Scientific, MA, Waltham, USA). FTIR spectra were also measured in Diffuse Reflection Infrared Spectroscopy (DRIFTS) mode with an Agilent 4100 ExoScan spectrometer (Santa Clara, CA, USA). This FTIR spectrometer equipped with an external reflectance probe allows measuring diffuse reflectance spectra of powders as well as of spherical beads without any specific preparation. In DRIFTS, when coming in to contact with the sample, the infrared light passes through the top surface of the sample before being reflected back out of the sample and into the detector of the spectrometer. Even if distortions from specular reflectance artifacts cannot be excluded, qualitative spectral comparisons could be done. The background was performed on a silicium wafer reference, the acquisition time was 30 s and the resolution was 4 cm^−1^.

#### 2.2.4. ICP-AES Analysis

The chemical nature of the 4A samples was determined through Induced Coupled Plasma—Atom Emission Spectroscopy (ICP-AES) on a Perkin Elmer Optima 2000 DV apparatus (Waltham, MA, USA).

## 3. Results and Discussion

### 3.1. Gas Porosimetry

#### 3.1.1. Argon Adsorption-Desorption at 87 K

**Figure 1:**
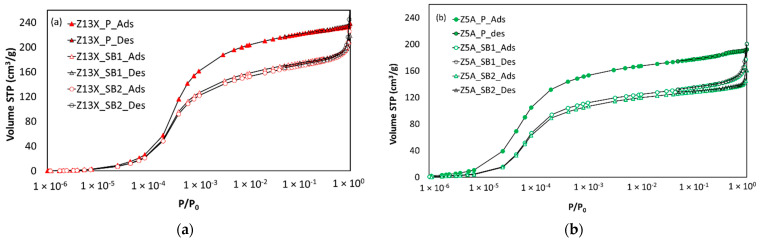
Argon adsorption–desorption isotherms at 87 K on different zeolites samples. (**a**) 13X zeolite, and (**b**) 5A zeolite.

Figure 1 compares the argon adsorption–desorption (fully reversible) isotherms at 87 K on the 13X and 5A zeolite samples throughout the entire relative pressure range and shows that the isotherms are fully reversible.

**Figure 2:**
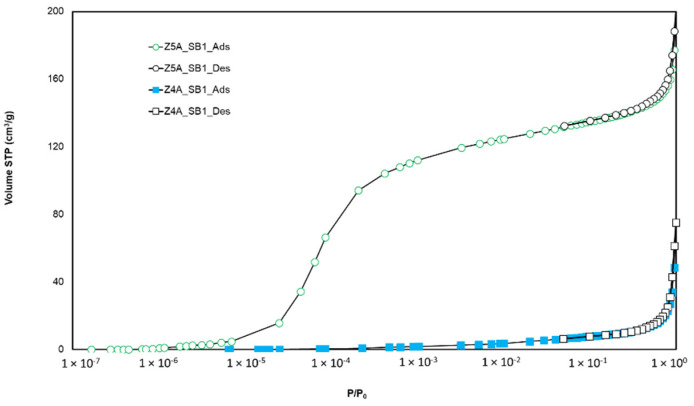
Comparison of argon adsorption–desorption isotherms at 87 K on 5A and 4A zeolites in spherical bed shape.

From Figure 2, it can be inferred that the 4A zeolite sample does not allow for the uptake of argon in its micropores, as significant diffusional limitations and molecular crowding phenomena are encountered for pore sizes lower than 4.5 Å for argon at 87 K [14,18].

**Figure 3:**
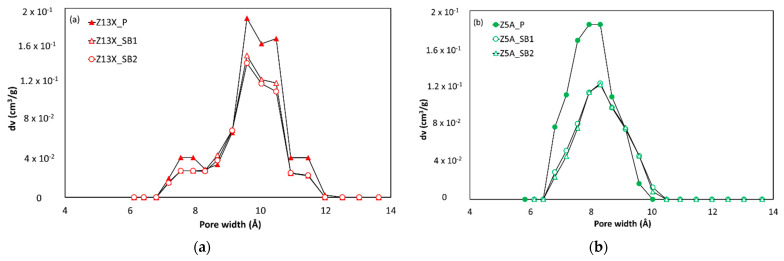
Pore size distribution of different zeolite samples. (**a**) 13X zeolites, and (**b**) 5A zeolites obtained with a NLDFT model (Quantachrome’s ASiQwin Software).

The Pore Size Distribution (PSD) and the micropore volume of each sample were obtained by applying the NLDFT model for argon adsorption on cylindrical/spherical pores of zeolites available in the Quantachrome’s ASiQwin Software. The BET surface area was calculated according to the procedure defined for microporous adsorbents by Rouquerol et al. [19]. These results are shown in Figure 3 and Table 2, respectively.

The adsorbed amount, the micropore volume, and the BET surface area have been measured on dry samples. The observed difference in Figure 1 and Table 2 between powder and spherical beads samples can be attributable to the presence of the binders contained in the shaped samples. The difference between powders and beads is the same for BET surface area as for the pore volume, which proves that the binder does not influence the porosity of the sample (pore blocking). The amount of binder may vary depending on the adsorbent manufacturer and can be calculated from the difference in adsorbate amounts, pore volumes, and BET surfaces between powder and shaped simples. Hence, from the differences between both the pore volumes and surfaces between the pure zeolites (powders) and the pelletized samples given in Table 2, the binder amount can be estimated between 22–24 wt% of the beads for 13X zeolites and to 24–28 wt% in the case of 5A zeolites, respectively. These results are in very good agreement with the literature [1,2,5].

The PSDs in Figure 3 are identical for both powder and spherical beads forms. Although 13X zeolite is composed of 7.4 Å windows and 13 Å cages [20], a single pore-filling mechanism is observed in the argon adsorption isotherm at 87 K (relative pressure interval between 10^−5^ and 10^−3^ approximately). That explains the obtaining of an “average” pore size of around 10 Å observed in the PSD. Likewise, 5A zeolite is composed of 5 Å windows and 11 Å cages [20] and the observed mean pore size in the PSD is around 8 Å. Besides, for spherical beads PSDs are similar, indicating almost the same micropore volume per unit of “porous” mass. According to the argon gas porosimetry results at 87 K, even if the spherical beads samples come from different providers, it is possible to state that the binder does not modify the adsorption properties of pure zeolites but logically impacts the surface area and micropore volume available to adsorption per unit mass of the sample. Finally, the more important argon uptake observed in the high relative pressure range (P/P0→1) indicates that the extrusion process leads to some degree of inter-particular meso- and/or macro-porosity.

#### 3.1.2. CO_2_ Adsorption–Desorption at 273 K

**Figure 4:**
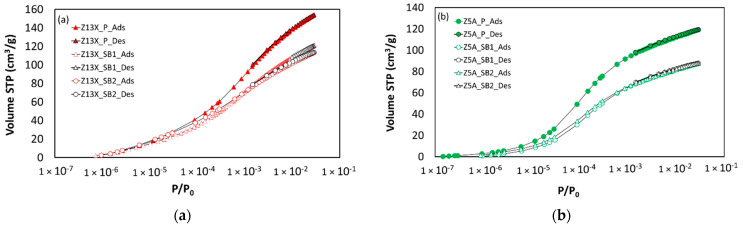
CO_2_ adsorption–desorption isotherms at 273 K on different zeolite samples: (**a**) 13X zeolites, and (**b**) 5A zeolites.

Figure 4 shows the CO_2_ adsorption–desorption isotherms at 273 K obtained for all the zeolite samples considered in this work. All the measurements were performed up to 101.3 kPa (i.e., P/P_0_ = 3.10^−2^).

CO_2_ isotherms at 273 K allow evaluating if the difference observed on powder and spherical beads from argon isotherms at 87 K is recovered or if the binder has an impact on the CO_2_ adsorption. In addition, in the case of some materials such as 4A zeolite in this work, CO_2_ adsorption is the only possibility to evaluate some structural key information using the gas porosimetry technique, as it is the only probe molecule able to enter the ultramicropores [18].

Regarding CO_2_ adsorption isotherms at 273 K for 13X and 5A zeolites (Figure 4), the difference in adsorbed volume between powder and granular beads samples is between 22 and 24 wt% in the case of 13X zeolite samples and between 24 and 28 wt% for 5A zeolite samples, respectively. Even if these zeolites contain different cations (Ca^2+^, Na^+^ for 5A zeolite and Na^+^ for 13X zeolite), the CO_2_ adsorption isotherms in powder samples are fully reversible and the gap observed in the argon adsorption isotherms between powder and shaped samples is recovered. Thus, one can conclude from the comparison between argon isotherms at 87 K and CO_2_ isotherms at 273 K that: (i) the binder present in spherical beads of these zeolites does not significantly influence the adsorption mechanism (regardless of the diffusional effects); (ii) the combination of Ar and CO_2_ porosimetry allows to quantify the binder amount; and (iii) the presence of different cations according to the considered zeolite does not change the two previous conclusions.

**Figure 5:**
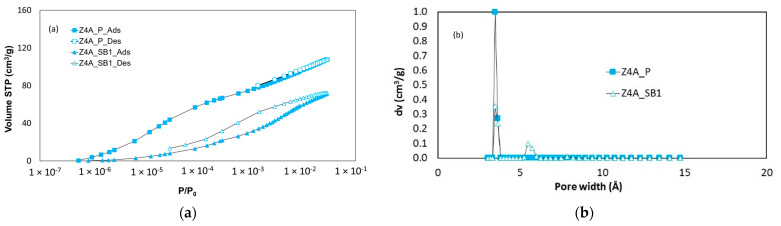
(**a**) CO_2_ adsorption–desorption isotherms at 273 K on 4A zeolite samples, and (**b**) Pore Size Distribution obtained with a NLDFT model. Adsorption and desorption branches are represented with different symbols in the case of the spherical beads of 4A zeolite to highlight the surprising irreversibility of the isotherm.

Yet in the case of the 4A zeolite (Figure 5a), the isotherm of CO_2_ is reversible in the case of the powder but irreversible in the case of the shaped beads. This fact suggests that the binder used to shape the beads of the 4A zeolite strongly interacts with the CO_2_ molecules. Furthermore, the CO_2_ uptake is shifted towards higher relative pressure values, indicating that the binders have an impact on the pore filling pressure and on the calculated PSD. Given the inexistence of available NLDFT models for zeolites in the Quantachrome’s ASiQwin Software, PSD was obtained by applying a NLDFT model for CO_2_ adsorption on carbon. Indeed, this model allows performing a qualitative analysis of the effect of the binder. From Figure 5b, one can conclude that the binder blocks part of the microporosity and induces an additional pore size, which leads to the observed shift of the filling pressure.

**Figure 6:**
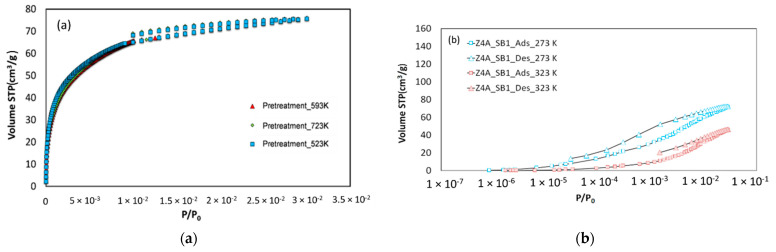
CO_2_ adsorption–desorption isotherms on 4A zeolite in spherical beads form. (**a**) At 273 K after pretreatment at different temperatures, and (**b**) At 273 K and 323 K.

To verify if the irreversibly of CO_2_ adsorption isotherm on 4A zeolite spherical beads samples was due to the pretreatment conditions, several isotherms were recorded with a pretreatment at different temperatures (523 K, 593 K, and 723 K) under secondary vacuum during 12 h. These results are shown in Figure 6a) and confirm that insufficient purification is not the cause of the irreversibility. This latter is also evidenced at a higher temperature (up to 323 K, see Figure 6b).

### 3.2. XRD Analysis

**Figure 7:**
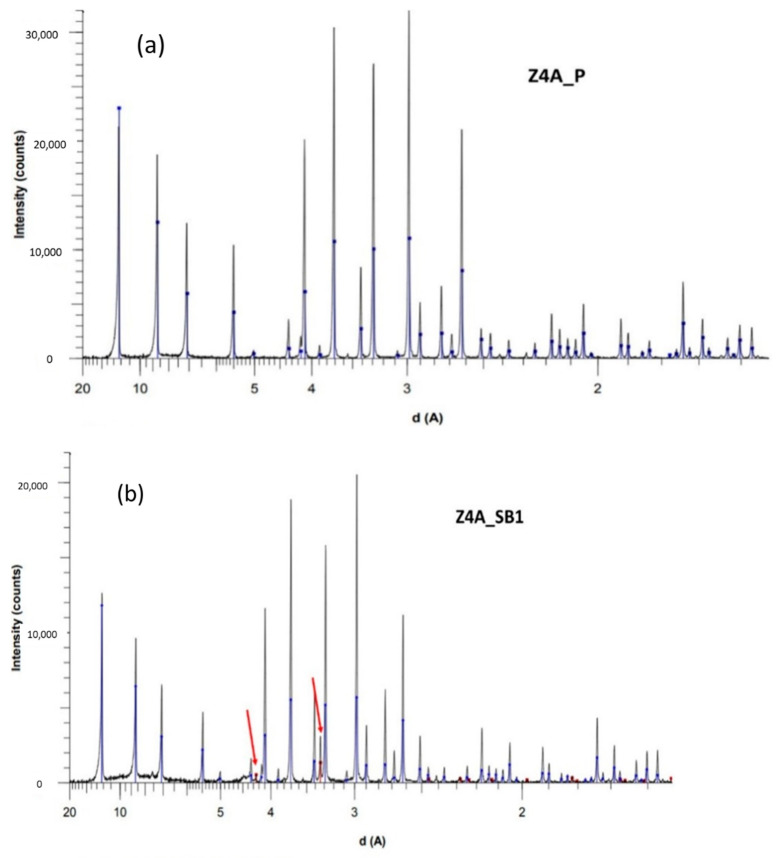
Comparison between X-ray diffractograms on the 4A zeolite samples. Experimental patterns of (**a**) the 4A powder samples, and (**b**) the 4A beads samples. Blue lines indicate the theoretical peak position of the ICDD PDF2 data file of the LTA zeolite, red lines indicate the theoretical peak position of the suggested SiO_2_ phase (highlighted by the arrow symbols).

The obtained diffractograms allow inferring all characteristic peaks for LTA and FAU phases (Figure 7 for 4A zeolite and supplementary information for 13X (Figure S1) and 5A (Figure S2) zeolites).

From the XRD patterns shown in Figure 7, additional small peaks attributed to a SiO_2_ phase are identified as quartz in spherical bead samples. There are two possible explanations for this occurrence: either there is some SiO_2_ from the synthesis left inside the zeolite pores or the binder itself contains quartz. In the first case, powder samples should contain this component too, hence the second hypothesis seems more likely. The presence of quartz could also partly contribute to the differences observed between powder and bead-shaped forms of zeolites on CO_2_ adsorption-desorption isotherms (Figure 1 and Table 2). No other differences are observed from XRD analysis.

### 3.3. Infrared Spectroscopy

**Figure 8:**
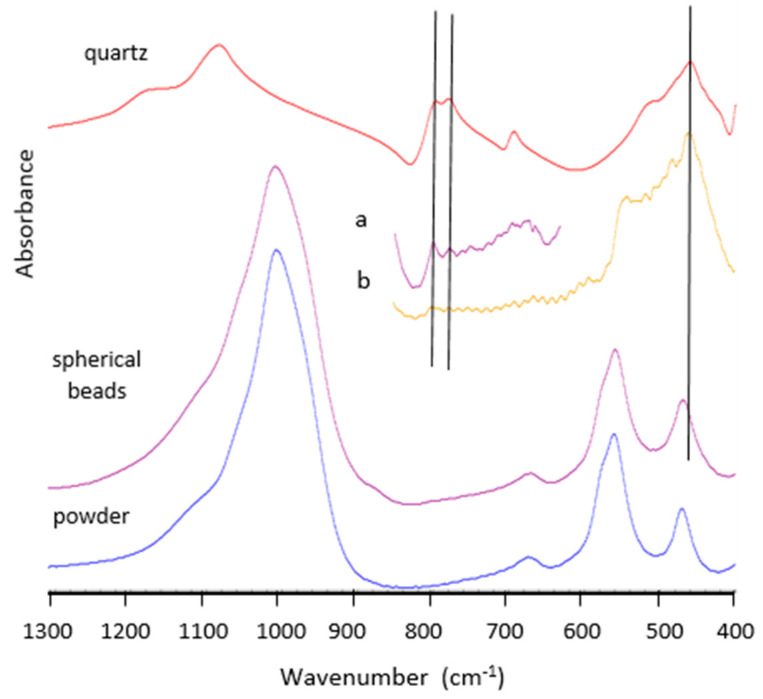
Transmission FTIR spectra of powder and powdered spherical beads for 4A zeolite samples (low concentration samples) and results of spectral subtractions (**a**) (high concentration samples) and (**b**) (low concentration samples) and comparison with quartz (HR Minerals library).

The FTIR spectra in the transmission mode of zeolite 4A as powder and ground spherical beads are given in Figure 8 and are very similar. The spectra subtraction (ground spherical beads minus powder) allows to evidence the occurrence of quartz in the spherical beads confirming the XRD data. Indeed, the characteristic doublet of quartz at 800 and 780 cm^−1^ is clearly observed after subtracting the spectra obtained for KBr pellets prepared with a high concentration of the sample (difference spectrum, a) in Figure 8. The difference spectrum obtained with a low concentration of the sample is also compatible with the presence of quartz in the spherical beads thanks to the band revealed at 460 cm^−1^ (b in Figure 8).

**Figure 9:**
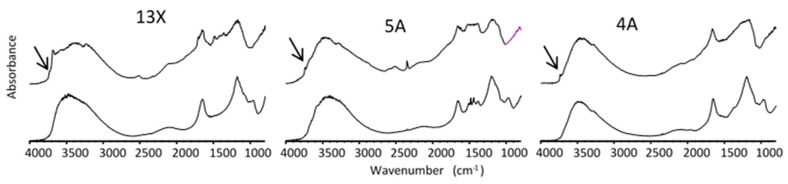
Comparison between DRIFT spectra on different 13X, 5A, and 4A zeolite samples as powders (**top**) or spherical beads (**bottom**). Arrows indicate the band at 3740 cm^−1^.

The DRIFT spectra for all zeolite samples are shown in Figure 9. DRIFT spectra reveal a hydrated silicate lattice for all zeolites. For all zeolite samples, a band at 3740 cm^−1^ (arrow on Figure 9) present for spherical beads and absent for powders indicates the occurrence of protonated sites at the surface of spherical beads.

### 3.4. ICP-AES Analysis

The chemical composition of the 4A samples obtained from ICP-AES analysis confirms the results obtained from the other techniques. Indeed, the chemical composition of the powder corresponds to the expected LTA zeolite, with a Na/Al ratio close to unity. The beads present substantially higher amounts of both Si and Na, which can clearly be inferred from the molar Si/Al and Na/Al ratios in Table 3. It is further interesting to note that in the powder sample some traces of calcium are present, but even this difference cannot explain the irreversibility of the pelletized 4A CO_2_ adsorption isotherm. These chemical compositions thus further confirm that the extruded samples were not prepared from the zeolite powder as starting material. The higher Si content in the beads compared to the powders are consistent with the detection of quartz in these samples using XRD and DRIFT techniques.

## 4. Conclusions

A control protocol based on gas porosimetry is proposed to assess the shaping effect on adsorption properties in shaped zeolites. The protocol consists of a combination of argon and CO_2_ adsorption at 87 K and CO_2_ at 273 K respectively. The comparison of the adsorption of an inert probe molecule such as argon on powdered and shaped samples allows quantifying the amount of binder and its effect on structural properties (pore volume, pore size distribution, and BET surface) for adsorbents with a pore size greater than 4.5 Å. The use of a molecule with a smaller kinetic diameter and a quadrupole moment such as CO_2_ allows determining the effect of the binder in adsorbents with smaller pore size, both on structural properties and adsorption mechanisms of the probe molecule.

The results show that the binder present in both 13X and 5A zeolites does not interact with CO_2_ and its content is approximately 25 wt%. However, the beads of 4A zeolite contain a binder that interacts with CO_2_ as the probe molecule. This effect is manifested by a shift in the CO_2_ pore filling relative pressures and the irreversibility of the adsorption isotherm. The irreversibility seems not to be sensitive to the pretreatment temperature (between 523 K and 723 K) nor the operating temperature (between 273 K and 323 K).

The results have been compared to other experimental techniques such as XRD, IR spectroscopy, and ICP-AES analyses. It was shown that the combination of these classical analytical techniques does not allow to fully identify the nature of the binders (which was not the objective of this work). Further investigations are needed for this purpose. Yet, overall results are very consistent, and the differences observed between the powders and shaped samples validate the control protocol proposed in this work.

## Figures and Tables

**Table 1:** Zeolite samples studied in this work.

Zeolite Sample	13X	5A	4A
Powder (P)	Z13X_P	Z5A_P	Z4A_P
Spherical beads 1 (SB1)	Z13X_SB1	Z5A_SB1	Z4A_SB1
Spherical beads 2 (SB2)	Z13X_SB2	Z5A_SB2	----

**Table 2:** BET surface area and micropore volumes of different zeolite samples.

Zeolite Sample	13X			5A		
	Z13X_P	Z13X_SB1	Z13X_SB2	Z5A_P	Z5A_SB1	Z5A_SB2
S_BE__T_ (m^2^/g)	799	624	607	641	482	461
V_p_ (NLDFT) (cm^3^/g)	0.37	0.28	0.28	0.35	0.25	0.23

**Table 3:** ICP-AES analysis results for the 4A zeolite samples.

Compound	Al (396.152 nm)		Na (396.152 nm)		Si (396.152 nm)		Ca (396.152 nm)		Molar Si/Al Ratio	Molar Na/AlRatio
	% mass	%RSD	% mass	%RSD	% mass	%RSD	% mass	%RSD		
Z4A_P	9.38	0.54	6.99	0.81	15.8	1.03	0.29	0.98	1.6	0.9
Z4A_SB1	5.56	3.14	11.4	0.60	17.2	0.58	-	-	3	2.4

## Data Availability

Data available on request.

## Supplementary Materials

The following are available online at https://www.mdpi.com/article/10.3390/nano11051205/s1, Figure S1. Comparison between X-ray diffractograms on the 5A zeolite samples. (a) shows the spectra from the 5A powder sample and (b) and (c) show the spectra from the 5A beads samples. Orange lines indicate the theoretical peak position of the ICDD PDF2 data file of the LTA zeolite, red lines indicate the theoretical peak position of the suggested SiO_2_ phase. Figure S2. Comparison between X-ray diffractograms on the 13X zeolite samples. (a) shows the spectra from the 13X powder sample and (b) and (c) show the spectra from the 13X beads samples. Magenta lines indicate the theoretical peak position of the ICDD PDF2 data file of the FAU zeolite, black points indicate the theoretical peak position of the suggested SiO_2_ phase.
