# Supplementary material for: Gas Porosimetry by Gas Adsorption as an Efficient Tool for the Assessment of the Shaping Effect in Commercial Zeolites
Source: Nanomaterials (Basel). 2021 May 1;11(5):1205. doi: 10.3390/nano11051205 (PMC8147277; doi:10.3390/nano11051205)
Supplement: Supplementary file 1 [file nanomaterials-11-01205-s001.zip › nanomaterials-1180500-supplementary.pdf]

Gas Porosimetry by Gas Adsorption as an Efficient Tool for the Assessment of the Shaping Effect in Commercial Zeolites

Alejandro Orsikowsky-Sanchez [1,2,*], Christine Franke [3], Alexander Sachse [4], Eric Ferrage [4], Sabine Petit [4], Julien Brunet [4], Frédéric Plantier [2] and Christelle Miqueu [2,*]

[1] TOTAL EP—Pôle d'Etudes et de Recherche de Lacq (PERL), BP 64170 Lacq, France;
[2] Laboratoire des Fluides Complexes et leurs Réservoirs, Université de Pau et des Pays de l'Adour,
E2S UPPA, CNRS, Anglet, 64600, France; frederic.plantier@univ-pau.fr (F.P.); christelle.miqueu@univ-pau.fr (C.M.);
[3] MINES ParisTech, PSL University, Center of Geosciences, 77305 Fontainebleau Cedex, France; christine.franke@mines-paristech.fr
[4] Université de Poitiers—IC2MP, UMR 7285 CNRS, 86073 Poitiers, France; alexander.sachse@univ-poitiers.fr (A.S.); eric.ferrage@univ-poitiers.fr (E.F.); sabine.petit@univ-poitiers.fr (S.P.); julien.brunet@univ-poitiers.fr (J.B.)
* Correspondence: christelle.miqueu@univ-pau.fr; alejandro.orsikowsky@total.com

Supplementary Material

5A powder and beads diffractograms

a) Z5A_P

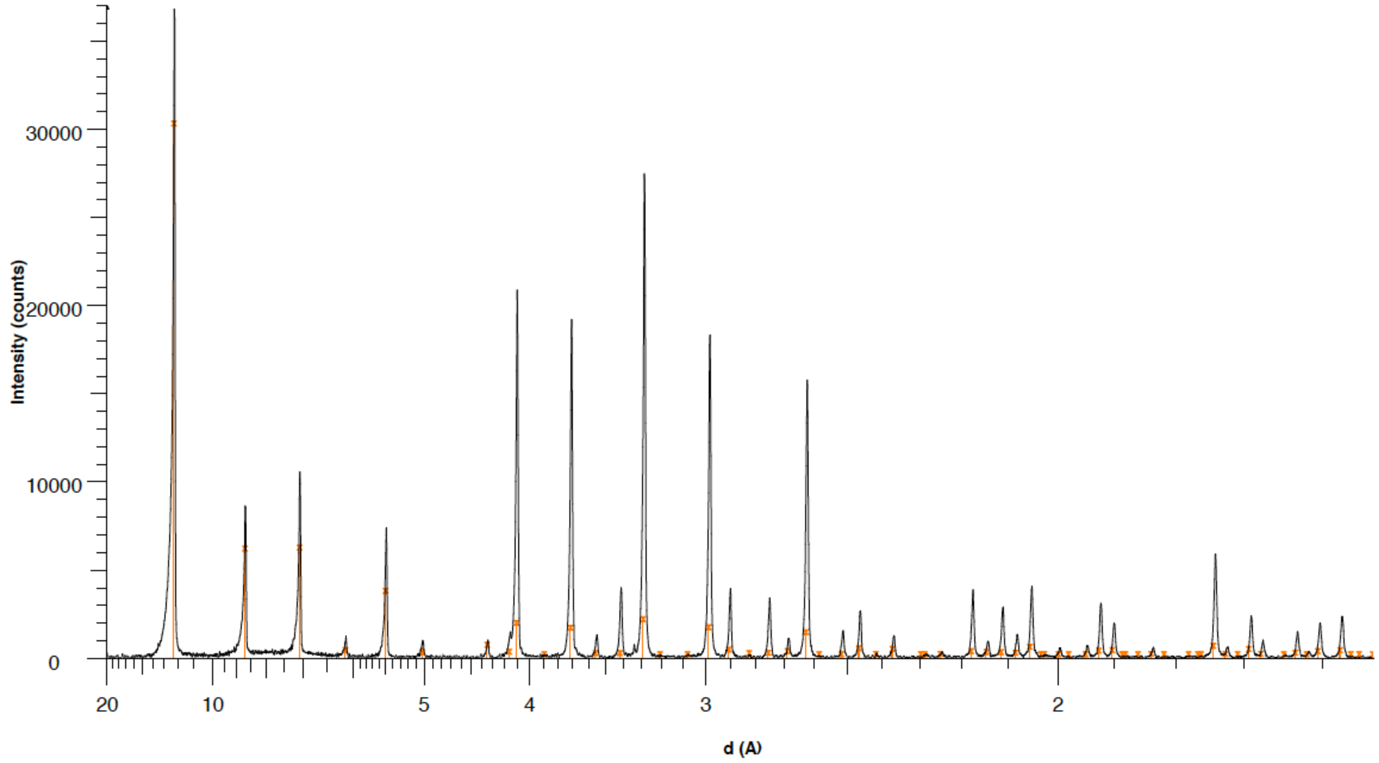

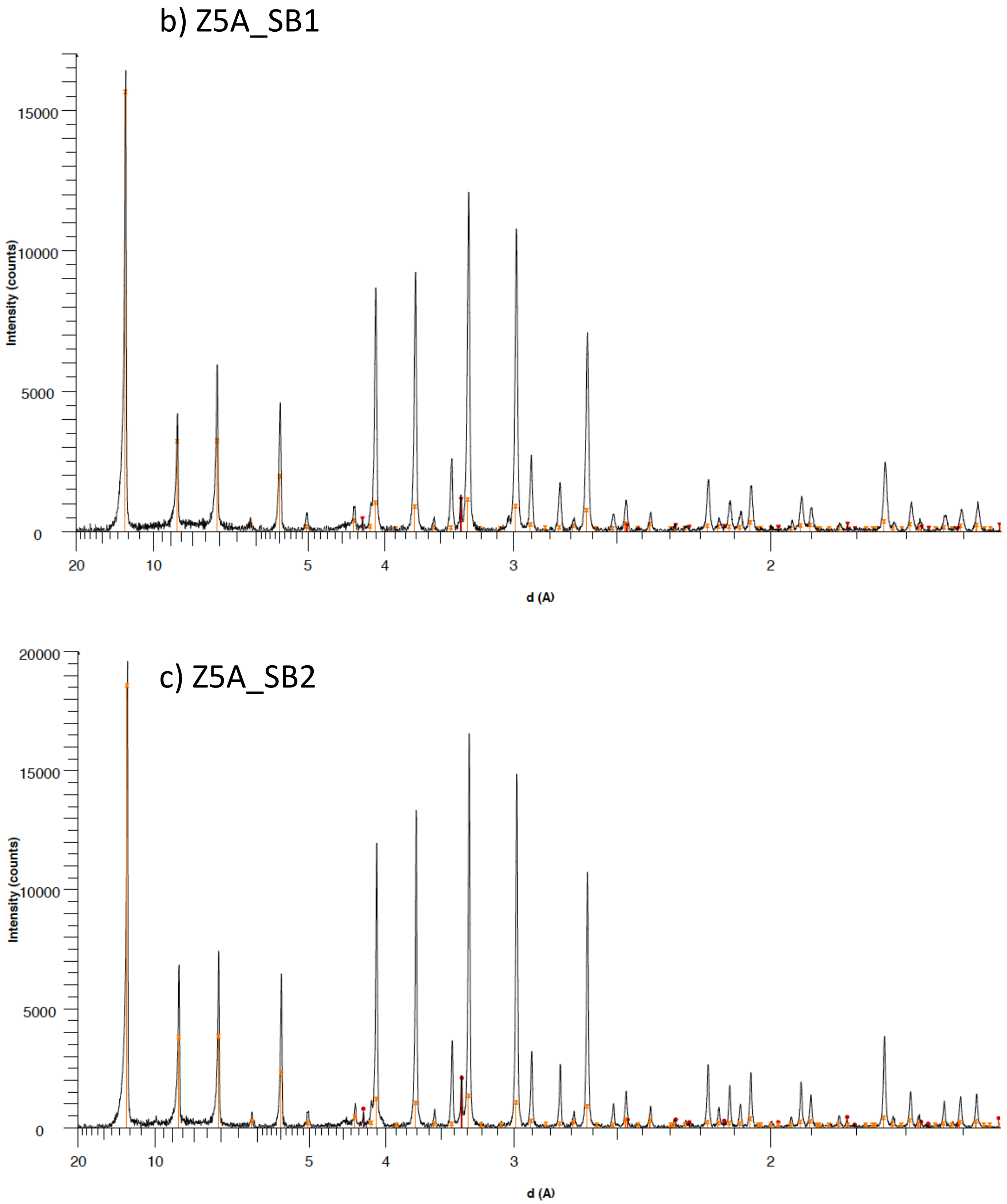

Figure S1. Comparison between X-ray diffractograms on the 5A zeolite samples. a) shows the spectra from the 5A powder sample and b) and c) show the spectra from the 5A beads samples. Orange lines indicate the theoretical peak position of the ICDD PDF2 data file of the LTA zeolite, red lines indicate the theoretical peak position of the suggested SiO_2 phase.

13X powder and beads diffractograms

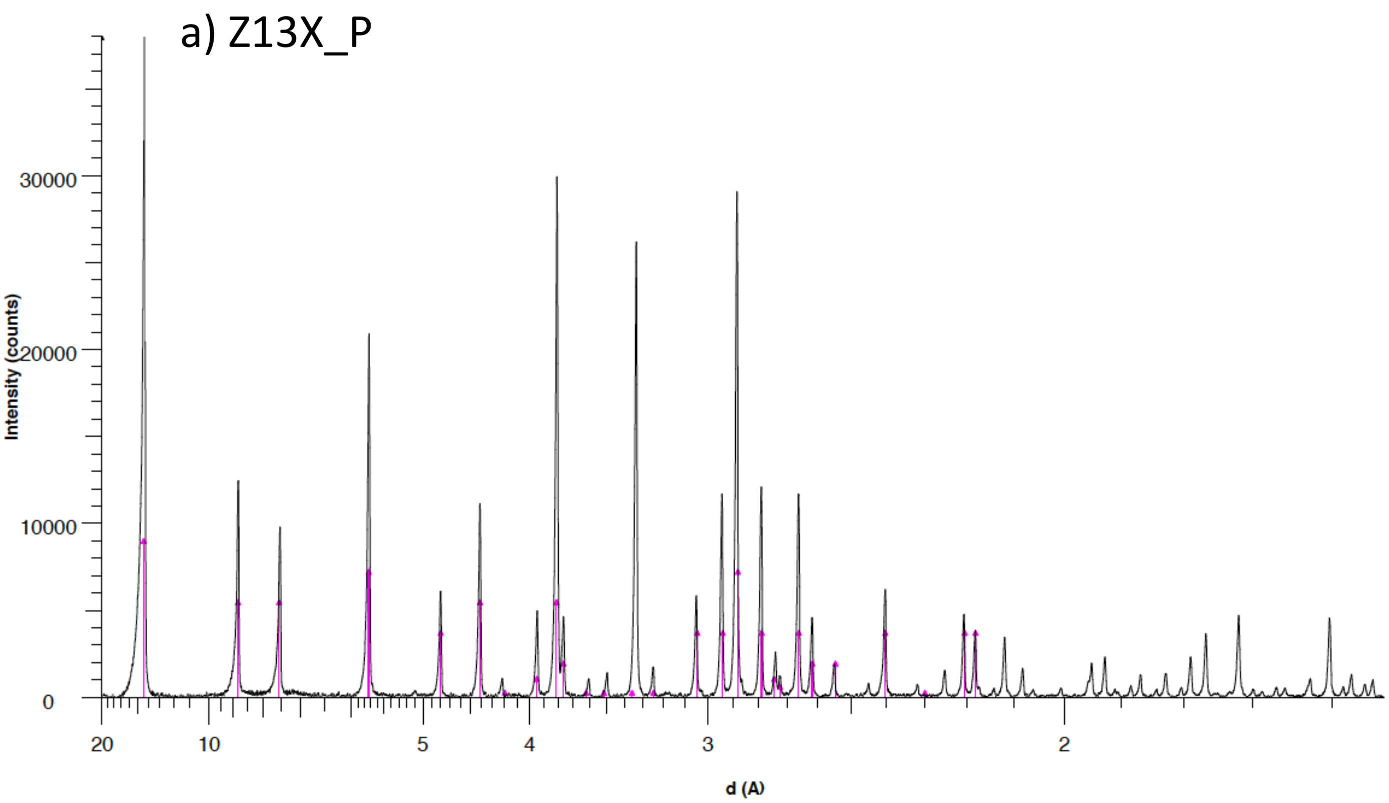

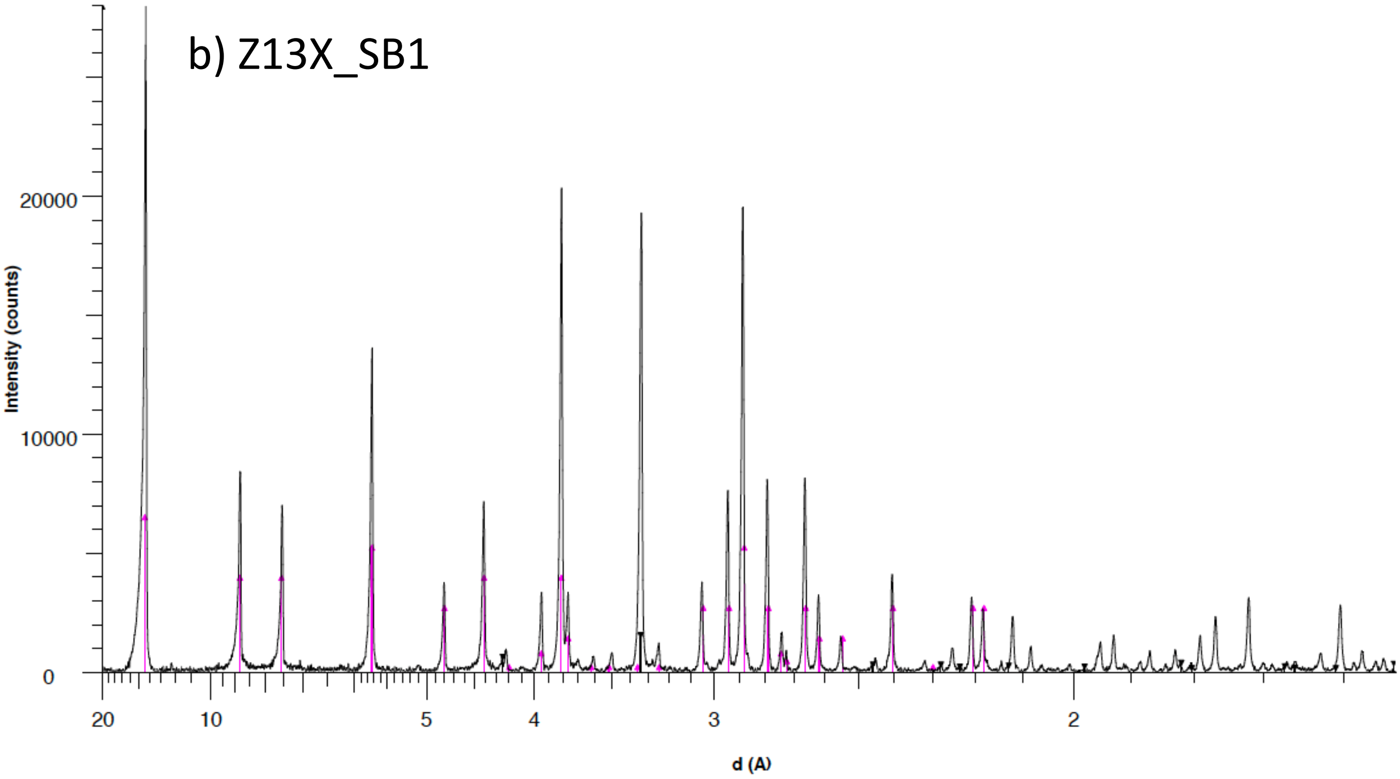

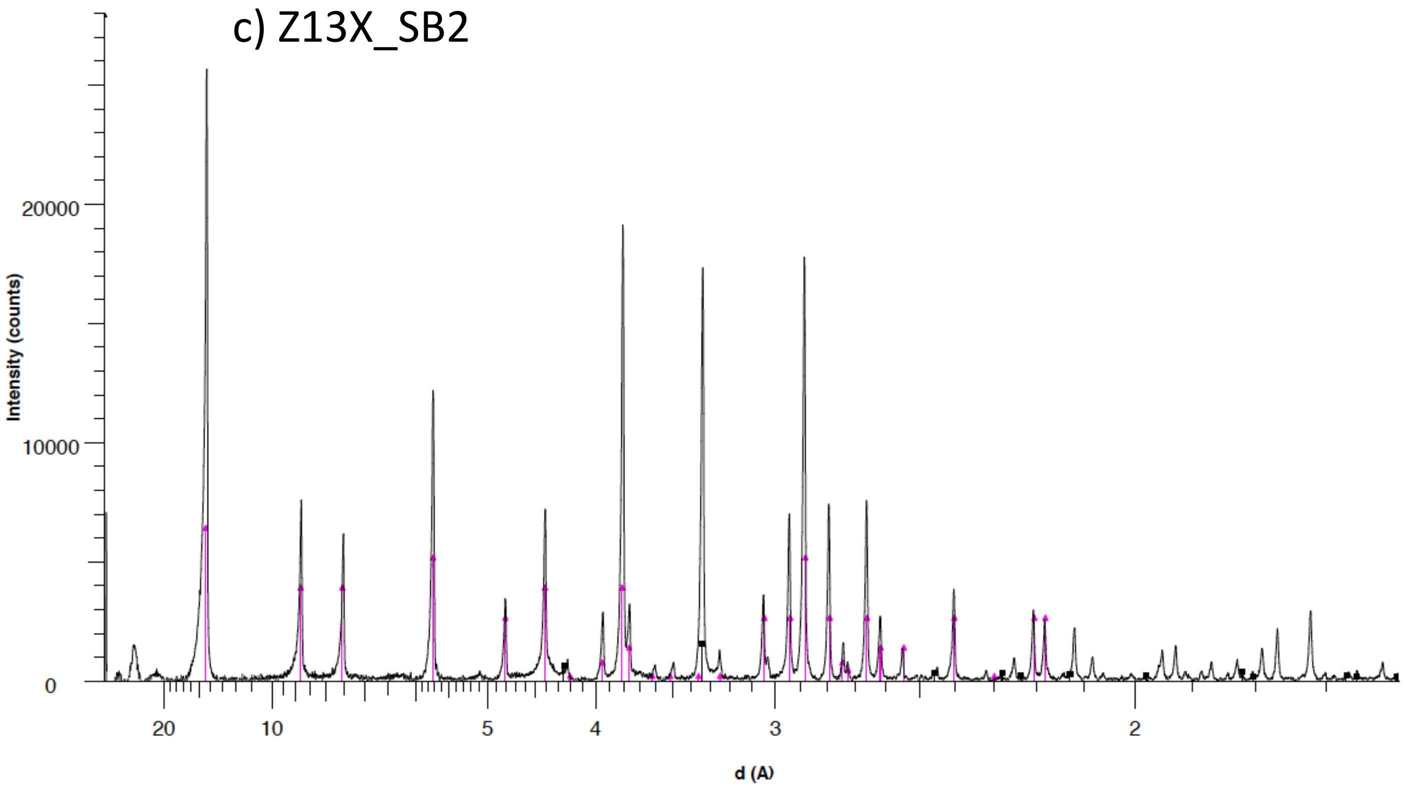

Figure S2. Comparison between X-ray diffractograms on the 13X zeolite samples. a) shows the spectra from the 13X powder sample and b) and c) show the spectra from the 13X beads samples. Magenta lines indicate the theoretical peak position of the ICDD PDF2 data file of the FAU zeolite, black points indicate the theoretical peak position of the suggested SiO2 phase.
